# Effective emotion regulation strategies improve fMRI and ECG markers of psychopathology in panic disorder: implications for psychological treatment action

**DOI:** 10.1038/tp.2015.160

**Published:** 2015-11-03

**Authors:** A Reinecke, N Filippini, C Berna, D G Western, B Hanson, M J Cooper, P Taggart, C J Harmer

**Affiliations:** 1 Department of Psychiatry, University of Oxford, Warneford Hospital, Oxford, UK; 2 Centre for Functional Magnetic Resonance Imaging of the Brain, University of Oxford, Oxford, UK; 3 Service d'anesthésiologie Centre Hospitalier, Universitaire Vaudois, Lausanne, Switzerland; 4 Department of Mechanical Engineering, University College London, London, UK; 5 Department of Mechanical Engineering, University of Bristol, Bristol, UK; 6 Isis Education Centre, University of Oxford, Oxford, UK; 7 Institute of Cardiovascular Sciences, University College London, London, UK

## Abstract

Impairments in emotion regulation are thought to have a key role in the pathogenesis of anxiety disorders, but the neurobiological underpinnings contributing to vulnerability remain poorly understood. It has been a long-held view that exaggerated fear is linked to hyperresponsivity of limbic brain areas and impaired recruitment of prefrontal control. However, increasing evidence suggests that prefrontal–cortical networks are hyperactive during threat processing in anxiety disorders. This study directly explored limbic–prefrontal neural response, connectivity and heart-rate variability (HRV) in patients with a severe anxiety disorder during incidental versus intentional emotion regulation. During 3 Tesla functional magnetic resonance imaging, 18 participants with panic disorder and 18 healthy controls performed an emotion regulation task. They either viewed negative images naturally (Maintain), or they were instructed to intentionally downregulate negative affect using previously taught strategies of cognitive reappraisal (Reappraisal). Electrocardiograms were recorded throughout to provide a functional measure of regulation and emotional processing. Compared with controls, patients showed increased neural activation in limbic–prefrontal areas and reduced HRV during incidental emotion regulation (Maintain). During intentional regulation (Reappraisal), group differences were significantly attenuated. These findings emphasize patients' ability to regulate negative affect if provided with adaptive strategies. They also bring prefrontal hyperactivation forward as a potential mechanism of psychopathology in anxiety disorders. Although these results challenge models proposing impaired allocation of prefrontal resources as a key characteristic of anxiety disorders, they are in line with more recent neurobiological frameworks suggesting that prefrontal hyperactivation might reflect increased utilisation of maladaptive regulation strategies quintessential for anxiety disorders.

## Introduction

Anxiety disorders are very common and disabling conditions that cause a particularly high economic burden,^1, 2, 3, 4^ and the problem remains that not all patients show stable benefits in response to first-line intervention approaches.^5, 6^ To improve treatments and their application, it is essential to define key mechanisms underlying anxiety disorders, as these are likely to represent important targets for treatment. Clinical models of anxiety propose that impairments in the regulation of negative affect have an important role in the pathogenesis of a disorder, as they contribute to exaggerated fear responses.^7, 8^ Following neurobiological accounts of emotion regulation, the processing of threat involves signalling in limbic brain regions such as the amygdala, a key area implicated in the fast automatic registration of threat, whereas successful downregulation of this response is thought to be associated with increased recruitment of prefrontal areas of cognitive control.^9, 10^ This is well supported by studies showing that in healthy volunteers, deliberate downregulation of negative affect is correlated with increased activation in medial and lateral areas of the prefrontal cortex (PFC), and that such activation dampens limbic signalling.^11, 12^ In contrast, anxiety disorders are proposed to be associated with hyperresponsivity of limbic brain areas and impaired recruitment of prefrontal control.^13, 14^

However, a number of novel findings suggest that while decreased allocation of lateral and ventral prefrontal resources seems to be an important characteristic of participants with non-clinical high trait anxiety or worry,^15, 16^ activation in these areas is more likely to be increased in clinical anxiety disorders during threat processing. In particular, studies have reported increased activation in the dorsal anterior cingulate cortex (ACC) and dorsomedial PFC (dmPFC) in specific phobia,^17^ social anxiety disorder,^18, 19^ panic disorder^20, 21^ and generalised anxiety disorder.^22^ Similarly, anxiety-specific threat processing has increasingly been associated with heightened activation in dorsolateral (dlPFC),^23, 24^ ventrolateral (vlPFC)^20, 21, 25^ and ventromedial PFC (vmPFC).^23, 26, 27^

Recent neurobiological accounts of anxiety disorders argue that, different from a view assuming reduced prefrontal cognitive control, prefrontal hyperactivation might reflect increased utilisation of dysfunctional regulation attempts in anxiety disorders.^28, 29^ This is in line with anxiety disorders being associated with the development of avoidance and safety strategies, such as escaping the anxiety-provoking situation, or mental distraction from the threat stimulus. These avoidance-based strategies are believed to have a key role in maintaining the disorder and are targeted during exposure-based cognitive-behaviour therapy (CBT).^30, 31^ Experimental research has confirmed that during the presentation of threatening images, anxious participants are more likely to use dysfunctional regulation strategies such as suppression or cognitive avoidance. In contrast, they are less likely to use adaptive, successful techniques such as positive reappraisal.^32, 33^ In further support of the idea of anxiety disorders being associated with an increase rather than a decrease in neural responses associated with affect regulation, CBT has been shown to lead to a decrease in responsivity in prefrontal brain areas usually implicated in cognitive control, such as the dlPFC and vmPFC.^24, 34, 35^

These results question whether anxiety involves increased or decreased engagement of prefrontal areas involved in cognitive control, and how such activation patterns are functionally connected with activation in limbic areas of the fear circuit. This study therefore aimed to assess regional neural correlates of emotion regulation in unmedicated patients with panic disorder compared with healthy volunteers, and functional connectivity between amygdala and prefrontal areas thought to be implicated in cognitive control. We therefore used a well-established emotion regulation paradigm^12, 36^ that allows assessment of neural responses to threat images during incidental emotion regulation where pictures are viewed naturally, and intentional emotion regulation where patients use previously learned strategies of cognitive reappraisal similar to those typically taught in CBT. Furthermore, we measured beat-to-beat heart-rate variability (HRV), a measure of autonomic innervation of the brain to the heart, as an additional indicator of emotion control.^37, 38^ Increased HRV in response to stressful stimuli reflects a dominance of the parasympathetic over the sympathetic influence and therefore successful emotion regulation.^39^ In line with this, reduced HRV has been reported in patients with anxiety disorders.^40, 41^ We hypothesised that during incidental emotion regulation, patients would draw on their maladaptive regulation strategies, reflected in increased PFC activation, ineffective downregulation of limbic activation and reduced HRV. In contrast, we expected these limbic–prefrontal activation patterns and HRV reductions to be dampened during intentional regulation, where alternative, adaptive control strategies would be used.

## Materials and methods

### Participants

Following *a priori* power calculations, 18 unmedicated patients with panic disorder (10 with/8 without agoraphobia) naive to psychological treatment and 18 healthy controls without Diagnostic and Statistical Manual of Mental Disorders (DSM-IV)^42^ axis-I history were recruited from the public (Table 1). Statistical power information was derived from behavioural data gained in a previous study using a faces dot-probe task in patients versus healthy controls.^43^ These calculations suggested that with an alpha level of 5%, sample sizes of 18 per group would be sufficient to gain statistical power of 80% (condition masked fearful faces: patients *M*=31, s.d.=54, controls *M*=−7, s.d.=35). Diagnoses were assessed using the Structured Clinical Interview for DSM-IV Axis I Disorders.^44^ Three patients fulfilled criteria for comorbid-specific phobia, with panic disorder being the primary diagnosis. General exclusion criteria were left-handedness, magnetic resonance imaging (MRI) contraindications, epilepsy, history of psychotic, bipolar or substance abuse disorder, and antidepressant treatment during the last 6 months. Three patients having reported occasional (but not regular) on-demand benzodiazepine or propranolol intake were medication-free 48 h before scanning. Ethical approval was obtained from the local research ethics committee.

### Mood and subjective state

Participants completed the Hospital Anxiety and Depression Scale,^46^ Body Sensations Questionnaire and Agoraphobic Cognitions Questionnaire.^47^ Before and after the scan, they completed Visual Analogue Scales for the dimensions anxious, sad, calm and happy (0–100 mm, not at all—extremely) to assess state mood.

### fMRI task design

Stimuli were 40 negatively valenced coloured IAPS images^48^ picturing characteristic panic-related catastrophic expectations, such as accidents or funerals (mean valence ratings 2.8±1.7, mean arousal ratings 6.0±2.2 on 9-point Likert scales ranging from 1=unpleasant/low arousal to 9=pleasant/high arousal). Valence and arousal ratings as well as scene content were matched between the two experimental conditions Maintain and Reappraisal. The order of picture blocks remained constant across all participants, with half of the subjects per group starting with a Maintain and half starting with a Reappraisal block.

Pictures were presented in eight blocks of five images, one after another for 5 s each, separated by 1-s blank screen interstimulus intervals. Picture blocks alternated with grey fixation baseline blocks of 30 s, and experiments started with a baseline block. For half of the blocks, participants were instructed to passively view the images and naturally experience the emotional state evoked, without attempting to regulate or alter it (Maintain blocks). For Reappraisal blocks, they were instructed to downregulate the provoked negative affect by using strategies of cognitive reappraisal (for example, reframing, rationalising). These strategies were trained before the scan using different images. Instructions were given by presenting the word Maintain or Reappraise on screen for 4 s before a block. At the end of each picture block, a 4-point rating scale (1=neutral, 4=negative) was presented for 4 s, and participants indicated the intensity of negative affect experienced throughout the block using a keypad. The total task duration was ~10 min.

### ECG recording

Throughout functional scanning, three-electrode electrocardiogram (ECG) was recorded to calculate HRV, separately for Maintain and Reappraisal blocks, using Siemen's PERU system (Erlangen, Germany). Signals were processed using custom algorithms.^49^ In brief, heart beats were timed at the instants of R-wave peaks in the ECG signal, and heart rate was measured as the inverse of consecutive R-wave to R-wave interval period. The timing of individual heart beats was automatically identified and manually corrected. The intervals between beats were calculated to construct a time series over the course of the experiment representing variations in the subject's heart rate. This series was then used to calculate low-frequency (LF; 0.04–0.15 Hz) and high-frequency (HF; 0.15–0.4 Hz) HRV parameters, using the spectral averaging technique.^50^ These parameters were calculated separately for Maintain versus Reappraisal blocks.

### Image acquisition

Images were obtained using a 3-T Siemens Sonata scanner. Functional imaging data were analysed using FEAT 6.0, part of FSL (FMRIB Software Library; fmrib.ox.ac.ul/fsl) with *Z*>2.3 and *P*<0.05, including multiple-comparison corrections. T_2_*-weighted functional data were acquired for a whole-brain field of view (64 × 64 × 40 matrix, 45 slices, voxel resolution 3 mm^3^, gap 1.5 mm, repetition time=3000 ms, echo time=30 ms, flip angle=90^o^). Field maps were acquired using a dual-echo two-dimensional gradient echo sequence with echos at 5.19 and 7.65 ms, and a repetition time of 500 ms. High-resolution T_1_-weighted images were acquired for subject alignment, using an MPRAGE sequence (174 × 192 × 192 matrix, voxel resolution 1 mm^3^, repetition time=2040 ms, echo time=4.7 ms, inversion time=900 ms).

### Image analysis

#### Event-related analysis

T_2_ pre-processing included motion correction,^51^ non-brain removal,^52^ spatial smoothing (Gaussian kernel full width at half maximum=5.0 mm), grand-mean intensity normalisation of the entire four-dimensional data set by a single multiplicative factor, registration of the functional space template to the anatomical space and the Montreal Neurological Institute (MNI) 152 space, highpass temporal filtering (Gaussian-weighted least-squares straight line fitting, with sigma=50.0 s), fieldmap correction. At the first-level, data were analysed using a general linear model approach with local autocorrelation correction.^53^ Two regressors of interest (Maintain, Reappraise) and two regressors of no interest (instruction/rating periods) were included. Fixation blocks were the implicit baseline reference. Contrast images were calculated for picture blocks in general, Maintain blocks, Reappraisal blocks, Maintain versus Reappraisal and Reappraisal versus Maintain. These individual activation maps were then entered into the group level (patients and controls), using a mixed-effects analysis across the whole brain.^54^ Due to strong evidence implicating the periamygdala region in threat processing using an almost identical task,^55^ region-of-interest (ROI) analyses were carried out for a 10-mm radius spherical mask around a previously published peak voxel (−14/−6/−8; and right-hemisphere counterpart).^12^ Significant whole-brain or ROI interactions were explored by (i) extracting BOLD signal changes within these areas and entering these into group × task mixed-design analyses of variance (ANOVAs) and appropriate follow-up *t*-tests, and (ii) running Pearson's correlation analyses for the percent signal change and panic symptom severity (calculated as the mean of the scores achieved on the Agoraphobic Cognitions Questionnaire and Body Sensations Questionnaire).

#### Connectivity analyses

We closely based this analysis on previous work showing that the onset of threat stimuli alters functional connectivity with an anatomy-based amygdala functional cluster.^56^ For each participant, we extracted a deconvolved time series for the functional cluster identified in the anatomical (i) right amygdala mask (peak: 26,0,−14; *Z*=6.3) and (ii) left amygdala (peak: −22,−4,−12; *Z*=6.7), in the pictures versus baseline contrast across groups, using small volume correction. These time courses were entered in two FSL psychophysical interaction analyses, separated for the functional right amygdala versus left amygdala cluster as seed region, along with the two psychological regressors (Maintain and Reappraisal), the two psychophysical interaction regressors (Maintain × time series and Reappraisal × time series) and the regressors of no interest (instructions and ratings). These individual contrast images were then entered into the group level, using a mixed-effects analysis across the whole brain, in order to identify brain areas that showed activity that covaried stronger with that of the left and right amygdala in one of the two groups during Maintain blocks, Reappraisal blocks or picture blocks in general. Pearson's correlations were computed for standardised betas (extracted from significant clusters) and panic symptom severity (calculated as the mean of the scores achieved on the Agoraphobic Cognitions Questionnaire and Body Sensations Questionnaire).

Given previous research indicating increased amygdala–dmPFC connectivity during the anticipation of threat^56, 57^ and in anxiety disorders,^58^ strength of coupling between the amygdala seed regions and the dmPFC was identified using a ROI approach. We extracted regression standardised beta values reflecting coactivation between the amygdala seeds and a 10-mm radius drawn around 12/42/54, a previously published peak voxel of a dmPFC cluster relevant in emotion regulation using an identical task and instructions,^12^ and entered these into group × task ANOVAs and Pearson's correlation analyses (panic severity).

#### Voxel-based morphometry

Voxel-based morphometry was carried out to be able to add grey matter maps as covariates to the functional MRI (fMRI) analysis model, to only identify group differences in functional activation that reflect cognitive-emotional rather than grey matter differences. Brain extraction and tissue-type segmentation were performed and resulting grey matter partial volume images were aligned to standard space using first linear (FLIRT) and then non-linear (FNIRT) registration tools. The resulting images were averaged, modulated and smoothed with an isotropic Gaussian kernel of 7 mm full width at half maximum to create a study-specific template, and the grey matter images were re-registered to this, including modulation by the warp field Jacobian. Threshold-free-cluster-enhancing correction was applied. Finally, voxel-wise general linear modelling was applied using permutation nonparametric testing (5000 permutations), correcting for multiple comparisons across space.

## Results

### Affect ratings and behavioural data

#### Mood and anxiety measures

Patients reported significantly higher trait anxiety and depression (Hospital Anxiety and Depression Scale) levels than controls, and they showed more fear of physical sensations (Body Sensations Questionnaire) (Table 1). With respect to state mood, there were significant group differences on all Visual Analogue Scales taken before and after the scan (Table 1). Patients showed lower anxiety scores and higher calm scores after the scan compared with baseline (both *t*>3.20, both *P*<0.005).

#### HRV and negative affect ratings during scan

Differences were analysed performing group × task mixed-model ANOVAs. In patients but not controls, LF/HF ratio was higher in Maintain compared with Reappraisal blocks (Table 1; group × task *F*=5.89, df=1/26, *P*=0.023, *d*=0.83; paired-samples *t*-test *t*=2.14, df=14, *P*=0.049). During Maintain blocks, patients showed higher LF/HF ratios than controls (independent samples *t*=2.09, df=26, *P*=0.047, *d*=0.82), suggesting reduced HRV and more sympathetic compared with parasympathetic activation. Groups were not significantly different in LF/HF ratio during Reappraisal (*t*=0.52, df=26, *P*=0.606).

Negative affect ratings were lower in Reappraisal versus Maintain blocks in both groups, without between-group differences (Table 1; Task *F*=45.26, df=1/34, *P*<0.001; group/group × task both *F*<0.04, both *P*>0.855).

### BOLD fMRI

#### Whole-brain analysis

**Figure 1:**
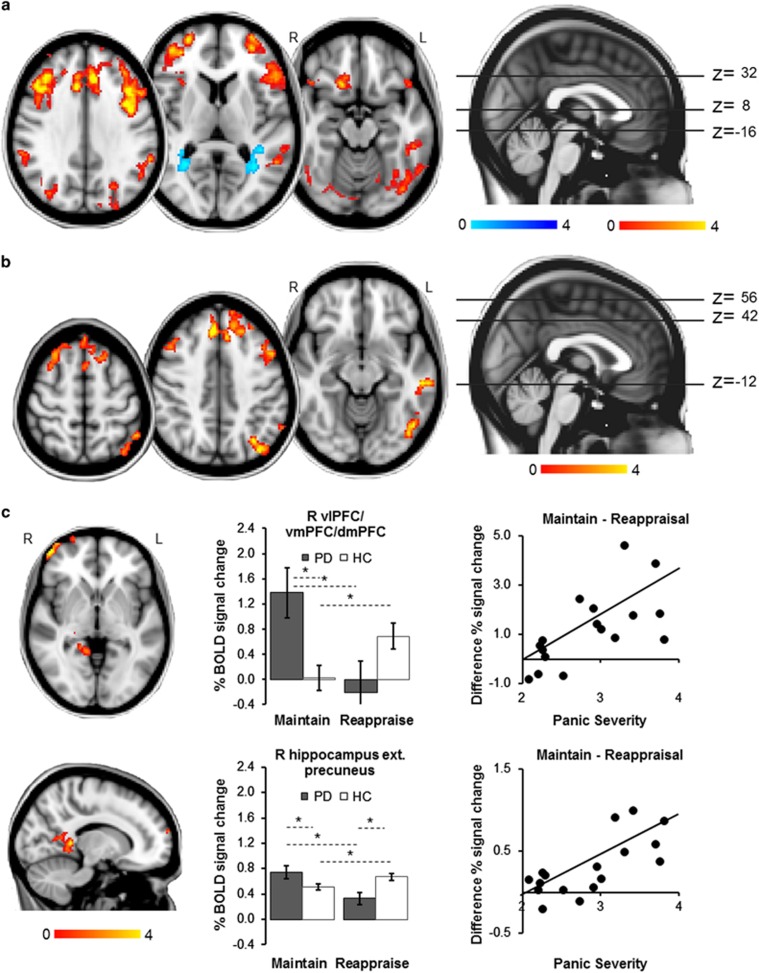
Whole-brain fMRI results. All images thresholded at *Z*>2.3, *P*<0.05, corrected. (**a**) Main effect of task: across both groups, Reappraisal (versus Maintain) led to greater BOLD signal response in bilateral dorsal anterior cingulate cortex, dorsomedial, dorsolateral, and ventrolateral PFC, orbitofrontal cortex, lateral occipital cortex, angular gyrus, cerebellum and occipital fusiform and inferior temporal gyri, and left middle temporal gyrus, and to a decrease in activation in bilateral precuneus ext. lingual gyrus. (**b**) Main effect of group: compared with controls, patients showed increased activation in prefrontal, temporal and occipital areas, including bilateral dorsomedial and dorsolateral PFC, left dorsal anterior cingulate cortex, right supplementary motor area, left inferior frontal and middle temporal gyri, left lateral occipital cortex and occipital fusiform gyrus, and left angular gyrus during picture blocks (versus fixation baseline block). (**c**) Group × task interaction: maintaining negative affect (versus Reappraisal) was associated with increased signal response in patients compared with controls in a right frontal pole cluster including the vlPFC, vmPFC and dmPFC (top panel), and a limbic cluster including parts of the right hippocampus, posterior cingulate cortex, precuneus and lingual gyrus (bottom panel). In controls, Maintain (versus Reappraisal) was related to decreased activation in this limbic cluster. For both clusters, BOLD% signal change during Maintain minus Reappraisal blocks was significantly correlated with symptom severity in patients. MNI coordinates 14,−42,−2. Error bars show s.e.m. *Significant difference between conditions or groups. dmPFC, dorsomedial PFC; ext., extending into; HC, healthy control; L, left; PD, panic disorder patient; PFC, prefrontal cortex; R, right; vlPFC, ventrolateral PFC; vmPFC, ventromedial PFC.

Main effect of task (Reappraise versus Maintain, across groups): Reappraisal was associated with increased activation in bilateral dorsal ACC, dmPFC, dlPFC, vlPFC, angular gyri, superior lateral occipital cortices, orbitofrontal cortices/subcollosal cortices/caudate, cerebellum/occipital fusiform/inferior temporal gyrus, and left middle temporal gyrus. Reappraisal also led to significant deactivation in bilateral precuneus/lingual gyrus (Figure 1a; Table 2A).

Main effect of group (picture blocks versus baseline): Compared with controls, patients showed significantly higher activation in bilateral dmPFC and dlPFC, left dorsal ACC and right supplementary motor area, as well as left inferior frontal gyrus, left middle temporal gyrus, left inferior and superior lateral occipital cortex, left occipital fusiform gyrus and left angular gyrus during the eight picture blocks versus the fixation screen baseline (Figure 1b; Table 2B; all *d*>1.19). Percent signal change in these clusters was not correlated with panic symptom severity in any of the groups (all *r*<0.26, *P*>0.293).

Group × task interaction (Maintain versus Reappraise): Results showed a significant group × task interaction in a frontal pole cluster spanning the right vlPFC and the right dmPFC and vmPFC, as well as a limbic cluster including parts of the right dorsal hippocampus and posterior cingulate, precuneus and lingual gyrus (Figure 1c; Table 2C). *Post hoc* analyses on BOLD signal change extracted from each of the two clusters indicated that the PFC interaction was driven by a group difference in response to pictures during Maintain blocks (ANOVA task × group *F*=14.79, df=1/34, *P*=0.001, *d*=1.32), with patients showing significantly higher activation than controls (Maintain *t*=2.77, df=34, *P*=0.009, *d*=0.80; Reappraise *t*=1.60, df=34, *P*=0.119, *d*=0.55). The group × task interaction in the hippocampal cluster was based on patients showing an increase in activation in this area in the Maintain condition and a decrease during Reappraisal (group × task interaction *F*=14.83, df=1/34, *P*<0.001, *d*=1.32; *post hoc t*-tests Maintain *t*=2.05, df=34, *P*=0.048, *d*=0.63; Reappraise *t*=2.93, df=34, *P*=0.006, *d*=1.14). In patients but not controls, BOLD activation in both these clusters during Maintain minus Reappraisal blocks was positively correlated with panic severity (PFC: patients *r*=0.49, *P*=0.038, controls *r*=0.36, *P*=0.142; hippocampus: patients *r*=0.49, *P*=0.038; controls *r*=−0.23, *P*=0.369). However, these do not survive conservative Bonferroni correction (that is, required *P*<0.025).

ROI analysis: A hemisphere × group × task ANOVA for the BOLD percent signal change extracted for the left (Maintain: patients 0.40±0.44, controls 0.15±0.17; Reappraise: patients 0.11±0.36, controls 0.19±0.22) and right amygdala (Maintain: patients 0.37±0.55, controls 0.13±23; Reappraise: patients 0.10±0.37, controls 0.17±0.24) spheres revealed a significant group × task interaction (*F*=6.82, df=1/34, *P*_Bonferroni_=0.026, *d*=0.90), without any laterality differences (all *F*<0.10, all *P*_Bonferroni_>0.998). This effect was driven by patients showing higher activation than controls in Maintain blocks (*t*=2.43, df=34, *P*_Bonferroni_=0.042, *d*=0.81). In patients (*r*=0.42, *P*=0.043) but not controls (*r*=−0.13, *P*=0.613), Maintain minus Reappraisal BOLD percent signal change in the amygdala was positively correlated with panic symptom severity.

#### Connectivity analyses

**Figure 2:**
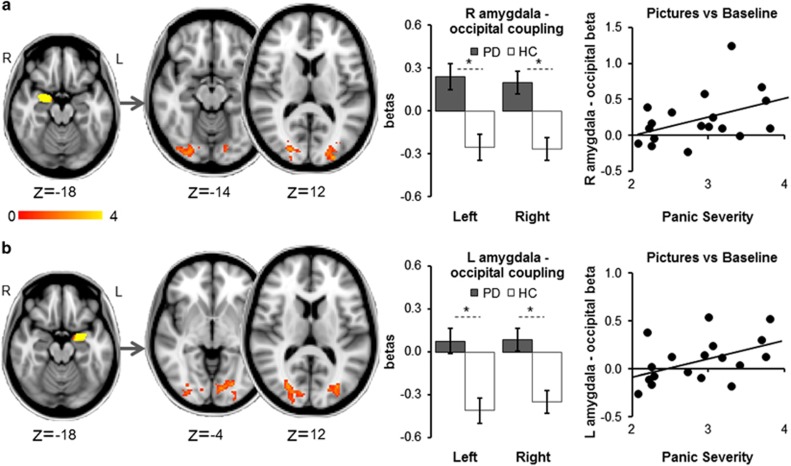
Whole-brain psychophysical interaction analysis with a right amygdala (**a**) and left amygdala (**b**) functional cluster (picture blocks versus baseline, across groups) as the seed region: patients showed higher connectivity of the right and left amygdala with left and right occipital pole, occipital fusiform gyrus, lateral occipital cortex and lingual gyrus. In patients, coupling between right amygdala seed and occipital cortex clusters and left amygdala seed and occipital cortex clusters was positively correlated with panic severity. Images thresholded at *Z*>2.3, *P*<0.05, corrected. *Significant difference between groups. Error bars show s.e.m. HC, healthy control; L, left; PD, panic disorder patient; R, right.

Whole-brain results: In patients versus controls, activity in each amygdala during picture blocks (versus baseline) was significantly more strongly correlated with activity in the left and right occipital cortices, occipital poles, occipital fusiform gyri and lingual gyri (right amygdala: right (R) cortical cluster: 591 voxels, MNI 14,−84,4, *Z*=3.86/left (L) cortical cluster: 511 voxels, MNI −10,−84,−4, *Z*=3.61; left amygdala: R cortical cluster: 578 voxels, MNI 28,−80,2, *Z*=3.67/L cortical cluster: 461 voxels, MNI −10,−84,−2, *Z*=3.47; all *d*>1.25). In patients but not controls, the magnitudes of right amygdala–occipital clusters and left amygdala–occipital clusters coupling were positively correlated with symptom severity (patients: L/R amygdala–occipital clusters both *r*>0.42, both *P<*0.042; controls: L/R amygdala–occipital clusters both *r*>0.35, both *P*>0.078) (Figure 2).

**Figure 3:**
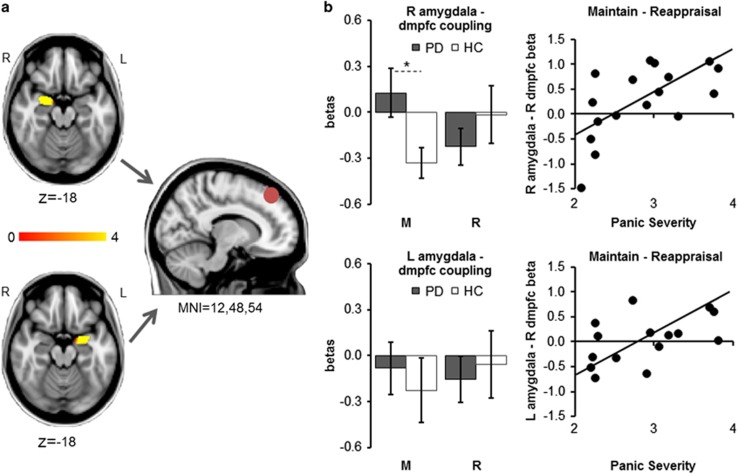
Psychophysiological interaction analyses exploring connectivity of activity within the right amygdala (**a**) and left amygdala (**b**) as seed regions and a right dorsomedial prefrontal cortex (R dmpfc) region of interest. The right amygdala showed a task × group interaction in connectivity with the dmpfc, with amygdala–dmpfc coupling being significantly greater in patients relative to controls during Maintain blocks. No such significant interaction was found for the left amygdala seed. In patients, higher right amygdala–dmpfc coupling, as well as higher left amygdala–dmpfc coupling during Maintain minus Reappraisal blocks were associated with higher panic severity. *Significant difference between groups. HC, healthy control; L, left; MNI, Montreal Neurological Institute; PD, panic disorder patient; R, right.

Amygdala–dorsomedial PFC coupling: For the right amygdala seed, we found a significant group × task interaction driven by patients showing higher right amygdala–right dmFC connectivity than controls during Maintain blocks (ANOVA group × task *F*=7.17, df=1/34, *P*_Bonferroni_=0.022, *d*=0.92; *t*-test *t*=2.39, df=34, *P*_Bonferroni_=0.046, *d*=0.80). For the left amygdala seed, the ANOVA yielded no group × task interaction (*F*=0.27, df=1/34, *P*_Bonferroni_=0.904). In patients but not controls, the magnitudes of right amygdala–dmPFC coupling and left amygdala–dmPFC coupling during Maintain minus Reappraisal blocks were positively correlated with panic symptom severity (patients: L/R amygdala–dmPFC clusters both *r*>0.55, both *P*<0.010; controls: L/R amygdala–dmPFC clusters both *r*<0.20, both *P*>0.214) (Figure 3).

Voxel-based morphometry: No group differences were observed in grey matter concentration. Furthermore, BOLD group contrast differences were not affected by adding individual grey matter maps as covariates to the fMRI analysis model, and they survived as clear differences between the two groups, suggesting that they reflect cognitive-emotional differences rather than being driven by sub-threshold grey matter differences between groups.

## Discussion

We believe this is the first study to simultaneously explore regional neurofunctional activation and limbic–prefrontal connectivity in patients with an anxiety disorder during incidental versus intentional emotion regulation. In line with our hypotheses, we found a pattern of increased brain activation in patients compared with controls in both limbic and prefrontal areas during incidental emotion regulation (Maintain) and in response to images in general. These differences were reduced, or even reversed, during intentional regulation (Reappraisal). The differences in brain activity were accompanied by significantly reduced heart rate variability in patients versus controls during incidental regulation only, highlighting patients' ability to regulate emotional response given appropriate cognitive strategies. Although these results challenge influential models of fear that propose impaired allocation of PFC resources as a neurobiological basis for the development of an anxiety disorder,^13, 14^ they are well in line with more recent frameworks.^28, 59^

In the neurobiological hypervigilance-avoidance model, Hoffman *et al.*^28^ postulate that threat processing in anxiety is associated with two different sets of functional activation patterns: hypervigilance processes and maladaptive, avoidant emotion regulation processes. The hypervigilance processes are thought to include amygdala hyperactivation in response to the detection of threat, which in turn facilitates visual processing in the occipital cortex. This is thought to enhance processes of selective attention and monitoring in the dmPFC while recruiting the hippocampus to provide information about memory associations with the potential threat stimulus.^28, 59^ The maladaptive regulation processes are thought to include hyperactivation in a range of ventral and lateral prefrontal–cortical regions known to be implicated in regulating negative emotional reactivity.

In line with the assumed hypervigilance circuit, our patients showed increased activation in occipital areas, dorsal mPFC and ACC, and increased amygdala–occipital connectivity when viewing threat images in general. They also demonstrated amygdala and hippocampus hyperactivation and increased amygdala–dmPFC connectivity during Maintain (versus Reappraisal) blocks. Most of these parameters correlated positively with symptom severity. These results also fit in well with other recent imaging studies, suggesting that threat processing in anxiety disorders is associated with increased activity in amygdala, hippocampus^21, 25, 26, 60, 61, 62^ and occipital cortex,^27, 62^ or in dorsal ACC and mPFC,^17, 18, 19, 20, 21, 22^ areas that have been implicated in selective attention, threat bias and monitoring.^56, 63, 64, 65, 66, 67, 68^

The results are also in line with prior studies demonstrating increased functional amygdala–dmPFC connectivity during the anticipation of threat in healthy volunteers,^56, 57^ with additional increases in subjects with higher trait anxiety, neuroticism or anxiety disorders.^56, 57, 58^ Furthermore, increased attentional bias magnitude derived from behavioural tasks has been shown to be correlated with increased amygdala–dorsal ACC connectivity in healthy volunteers,^69^ providing further evidence that such brain activation patterns might predispose patients to selectively focus their attention to threat information in their environment. The potential role of these proposed areas of hypervigilance in the psychopathology of anxiety disorders is further supported by clinical research showing a reduction of activation in amygdala,^17, 70^ hippocampus,^24^ dorsal mPFC and ACC^17^ following successful CBT.

The assumption that hyperactivation in lateral and ventral prefrontal–cortical regions might reflect anxiety-specific dysfunctional regulation strategies is supported by our findings of patients showing increased activation in dorsal and ventral lateral PFC in response to threat images in general. Furthermore, they showed increased response in vmPFC and vlPFC during incidental regulation, with activation strength correlating positively with panic severity. Lateral prefrontal activation has previously been implicated in intentional emotion regulation^11, 71, 72^ and inhibitory control,^73, 74^ and the vmPFC has particularly been associated with automatic conflict and emotion regulation^75, 76, 77^ in healthy volunteers. In line with our observations, other imaging studies exploring threat processing in anxiety disorders have reported heightened activation in the dlPFC,^23, 24^ vlPFC^20, 21, 25^ and vmPFC,^23, 26, 27^ and CBT has been shown to significantly reduce such hyperactivation.^34, 35^

Taken together, our results provide evidence in favour of recent models of anxiety, which propose that psychopathology might be underpinned by hyperactivation in both limbic and prefrontal–cortical brain regions in response to threat.^28, 59^ Such findings contradict previous frameworks postulating that impaired emotion regulation in anxiety is correlated with reduced recruitment of PFC areas of top–down control.^13, 14^ Strikingly, these early models were greatly based on research investigating neural processing in high trait anxiety or post-traumatic stress disorder (PTSD), which appear to characteristically be associated with decreased recruitment of PFC resources during threat processing.^29^ Neurobiological activation patterns in high trait anxiety in response to threat might still be adaptive and as such distinctive from activation patterns in anxiety disorders. Notably, PTSD is not classified as an anxiety disorder in DSM-V (APA, 2013) anymore, as the key symptom is the re-experience of a *de facto* trauma rather than arbitrary fear, and as fear is not the only and not necessarily the predominant emotion.^78, 79^ It appears plausible that these differences in aetiology and symptomatology between PTSD and anxiety disorders might be underpinned by differences in neurobiological pathophysiology, and future research will have to address these issues explicitly. It is also possible that differences in paradigms used between studies might contribute to contrary findings in prefrontal–cortical activation, with previous studies in trait anxiety often tapping into rapid resolution of emotional conflict^13^ rather than emotion processing and regulation *per se*.

It might appear puzzling why we rarely found group differences in functional activation for reappraisal blocks. However, reappraisal is one of the key strategies taught during CBT^80^ to which patients with panic disorder have been shown to be particularly responsive, even after only one treatment session.^81^ It is possible that having trained patients to successfully use reappraisal before scanning has provided them with sufficient, healthy regulation strategies. Furthermore, our results allow no final conclusions with respect to the exact role of the panic-specific processing patterns observed here in the onset and maintenance of an anxiety disorder. Future studies will have to establish whether these patterns of activity are sensitive to treatment, and whether any changes in these parameters are causally related to clinical improvement, which would confirm their proposed role as key mechanisms in the pathogenesis of anxiety.

## Figures and Tables

**Table 1:** Socioeconomic, mood and anxiety self-report, negative affect ratings and heart-rate variability scores in the two groups (mean±s.d., independent-samples *t*-test/*X* ^2^-test *P*-scores)

	*Panic patients*	*Healthy controls*	P-*score*
*Sociodemographic data*			
Age (years)	36.5±13.8	32.3±12.1	0.341
Gender	14 female/4 male	14 female/4 male	0.655
Years of education	16.6±2.7	17.5±4.2	0.473
Verbal IQ (NART^45^)	117.8±5.1	118.8±3.9	0.526

*Depression and trait anxiety*			
HADS—anxiety	14.6±4.1	2.0±1.6	<0.001
HADS—depression	8.1±3.4	0.7±1.0	<0.001

*Panic symptoms*			
BSQ	3.4±0.7	1.4±0.4	<0.001
ACQ	2.4±0.6	1.1±0.4	<0.001
			
*Heart rate variability*	*N=15*	*N=13*	
LF/HF Maintain	2.8±2.2	1.4±1.0	<0.047
LF/HF Reappraisal	2.0±1.6	1.7±1.1	NS

*Negative affect ratings*			
Maintain	2.8±0.6	2.9±0.6	NS
Reappraisal	1.9±0.6	1.9±0.7	NS

*State mood pre MRI scan*			
Anxious	67.7±23.5	4.3±6.1	<0.001
Sad	36.6±28.9	7.7±15.2	0.001
Calm	34.7±23.5	85.0±11.2	<0.001
Happy	49.3±24.4	75.3±12.8	<0.001

*State mood post MRI scan*			
Anxious	29.9±28.1	9.0±21.6	0.017
Sad	31.2±22.7	6.1±10.9	<0.001
Calm	54.3±27.4	79.7±18.7	0.003
Happy	53.1±20.3	70.8±17.3	0.008

Abbreviations: ACQ, Agoraphobic Cognitions Questionnaire; BSQ, Body Sensations Questionnaire; HADS, Hospital Anxiety and Depression Scale; IQ, intelligence quotient; LF/HF, low-frequency/high-frequency heart-rate variability ratio; MRI, magnetic resonance imaging; NART, National Adult Reading Test.

**Table 2:** (A) Areas of significant increase and decrease in BOLD response during voluntary emotion regulation (Reappraisal versus Maintain) across both groups. Areas of significant increase in BOLD response in patients versus controls during (B) picture blocks versus fixation baseline blocks, and (C) Maintain versus Reappraisal blocks. MNI coordinates refer to the peak activation voxel of the cluster and main sub-regions within the same cluster (significant group differences are in bold)

	*BA*	*Side*	*Cluster size (voxels)*	*MNI (x, y, z)*	Z*-score*	P*-score*
*(A) Main effect of task across groups*						
Increased activity during reappraisal (*R>M*)						
Dorsal ACC ext. dorsomedial PFC	**6/8/32**	**L**	**14 701**	**−4,20,50**	**5.86**	**<0.001**
Dorsal ACC ext. dorsomedial PFC	6/8/32	R		4,28,40	4.99	
Ventrolateral ext. dorsolateral PFC	9/45/46	R		36,46,12	4.9	
Cerebellum ext. occipital fusiform/inferior temporal gyri	**19/37**	**L**	**7 274**	**−36,−64,−28**	**4.30**	**<0.001**
Middle temporal gyrus	20/21	L		−60,−32,0	4.2	
Angular gyrus	39/40	R		44,−60,42	4.18	
Superior lateral occipital cortex	5/7	R/L		0,−68,68	4.06	
Angular gyrus	39/40	L		−60,−44,34	4.06	
Cerebellum ext. occipital fusiform/inferior temporal gyri	**19/37**	**R**	**2 184**	**36,**−**60,**−**52**	**4.46**	**<0.001**
Frontal orbital cortex	**11/38**	**R**	**544**	**16,22,**−**16**	**3.92**	**0.006**
Subcallosal cortex ext. caudate	25	R		6,20,−12	3.89	
Subcallosal cortex ext. caudate	25	L		−4,8,−10	3.29	
Decreased activity during reappraisal (*M>R*)						
Precuneus ext. lingual gyrus	**19**	**R**	**477**	**26,−32,22**	**3.67**	**0.014**
Precuneus ext. lingual gyrus	**19**	**L**	**434**	**−20,−42,24**	**3.90**	**0.024**
						
*(B) Pictures vs baseline/patients vs controls*						
Dorsal anterior cingulate cortex	**32**	**L**	**1 352**	**−8,28,38**	**3.99**	**<0.001**
Dorsomedial PFC	8/9	L		−24,38,46	3.65	
Dorsomedial PFC	8/9	R		4,42,46	3.56	
Supplementary motor area	6	R/L		4,14,72	3.55	
Dorsolateral PFC	**45**	**L**	**799**	**−46,30,28**	**3.90**	**<0.001**
Inferior frontal gyrus	44/48	L		−54,16,14	3.88	
Dorsolateral PFC	46	L		−40,34,26	3.65	
Dorsolateral PFC	9	L		−42,18,44	3.50	
** **Middle temporal gyrus ext. inferior lateral occipital cortex	**20/21**	**L**	**535**	**−56,−28,−12**	**3.77**	**0.003**
Occipital fusiform gyrus	37	L		−48,−66,−12	3.53	
Inferior lateral occipital cortex	19	L		−44,−74,−14	3.33	
Superior lateral occipital cortex	**7**	**L**	**493**	**−32,−68,40**	**3.97**	**0.006**
Angular gyrus	40	L		−50,−52,54	3.61	
Dorsomedial PFC	**9**	**R**	**459**	**20,36,54**	**3.30**	**0.009**
Dorsolateral PFC	44	R		50,18,38	3.28	
Dorsolateral PFC	8/9	R		42,18,50	3.19	
						
*(C) Maintain vs reappraise/patients vs controls*						
Ventrolateral PFC	**46/47**	**R**	**417**	**48,56,−2**	**3.98**	**0.030**
Ventromedial/dorsomedial PFC	10/11	R		32,68,2	3.15	
Hippocampus ext. precuneus/posterior cingulate	**27/29/30/37**	**R**	**387**	**14,−42,4**	**3.80**	**0.044**
Hippocampus	27	R		24,−34,−12	3.65	
Lingual gyrus	37	R		26,−42,−8	3.16	
Precuneus	30	R		12,−52,10	3.04	

Abbreviations: ACC, anterior cingulate cortex; BA, Brodmann area; ext., extending into; L, left; *M*>*R*, Maintain versus Reappraisal; MNI, Montreal Neurological Institute; PFC, prefrontal cortex; R, right; *R*>*M*, reappraisal versus maintain.
